# The Military Surgeon

**Published:** 1907-03

**Authors:** 


### The Military Surgeon

THE official organ of the Association of Military Surgeons, published by Major James Evelyn Pilcher, will hereafter be known as "The Military Surgeon," a change in title which is more distinctive of the character of the publication and one which appears more generally acceptable. Beginning merely as the channel through which the transactions of a limited association were given to the profession, it has grown under Major Pilcher's able editorship to be the one accepted authority on military medicine, hence its change of title gives it the proper recognition to which it is rightfully entitled.

Military medicine is a branch which is sadly neglected in the professional schools of this country; it does not appear to be considered in the light of a specialty, apparently, even in face of the mistakes and lessons of the Spanish war. However able a surgeon or physician may be he is a mere tyro when, without experience, he enters the field of military medicine, and for this reason may be ascribed the enormous paper losses sustained by the government during the recent war. Aside from the purely executive function of the military surgeon, the practice of medicine under arms is quite a different matter from the pursuit of the profession in civil life; and while it may be strictly termed a specialty, yet it is one in which the general practitioner will find much to interest him and much which will tend to improve his workaday methods.

Dr. Pilcher, as a pioneer in this field. and as editor of The Military Surgeon, has done work of inestimable value in placing before the profession the specialty of military medicine, and doing so in a manner which strips it of all the dry-rot exclusiveness characteristic of some of the other branches of practice. The Military Surgeon is interesting not alone to those who are closely allied to military medicine, but will be of benefit to others whose interest may be merely that of transient curiosity.

There is no country in the world which is not represented in the membership of the Association of Military Surgeons and the contributions to the pages of The Military Surgeon are of necessity varied, geographically speaking.

Membership in the Association is open to surgeons of the Army, Navy, National Guard, Naval Militia, Public Health and Marine Hospital Service, and to those who have ever had professional connection with any of these branches of the service. It is the duty of everyone who is eligible to assist in the upbuilding of a great organisation by giving it actual support. At any rate, a broad-minded interest in the welfare of the association would be personally beneficial to those who are not eligible to membership, either active or associate. Major Pilcher may be addressed at Carlisle, Pa.

